# Association between Second-Time Mother’s Prenatal Depression and Firstborn’s Behaviour Problems: The Mediation Role of Parenting Daily Hassles

**DOI:** 10.3390/ijerph182312794

**Published:** 2021-12-04

**Authors:** Ran Zhuo, Gendao Li

**Affiliations:** 1 School of Humanities, Jilin Agricultural University, Changchun 130118, China; zhuoran@jlau.edu.cn; 2 Department of Marketing, Operations and Systems, Newcastle Business School, Northumbria University, Newcastle upon Tyne NE1 8ST, UK

**Keywords:** behaviour problems in children, prenatal depression, parenting daily hassles, second-time mother, family system

## Abstract

Background: With the relaxation of birth control policy in China in recent years, second-time mothers’ mental health has raised concerns. However, the impact of firstborn children’s behaviour problems on second-time mothers’ prenatal depression in families transitioning to siblinghood has received little attention from family psychologists. Aims: This research aims to investigate whether firstborn children’s behaviour problems affect second-time mothers’ prenatal depression and the mediation role of daily parenting hassles, i.e., minor stressors associated with parenting, on this relationship. Methods: Data about second-time mothers’ prenatal depression, parenting daily hassles, and firstborn children’s behaviour problems were collected from 105 families transitioning to two children families using mother-reported questionnaires. Regressions were used to analyze the data. Results: About half of the mothers in the sample have depressive symptoms. Firstborns’ behaviour problems did not have a direct effect on the mother’s prenatal depression, but the problems did have an indirect effect via parenting daily hassles. The mothers’ age was significantly associated with prenatal depression. Conclusions: The mediation role of parenting daily hassles in the association with firstborn’s behaviour problems and mother’s prenatal depression suggests the need for support that reduce the levels of daily parenting hassles from firstborn children.

## 1. Introduction

Maternal prenatal depression is a major depressive disorder during pregnancy with symptoms such as sadness or low mood, appetite change, suicidal ideation, feelings of worthlessness, loss of interests, and so on [1]. Research has reported different proportions of prenatal depression: 11.9% in [2], 7–20% in high-income countries, and 20% or more in low-income and middle-income countries in [3]. Although the exact percentage varies in different research using different samples, the increasing prevalence of prenatal depression in the younger generation can be confirmed [4]. 

In China, the central government relaxed the birth control policy from “one-child policy” to “two-child policy” in 2016 and to “three-child policy” in 2021. This resulted in more and more families having two children in recent years, and firstborn children and mothers are facing great challenges due to the changes caused by the second child. This adaptation difficulty is not uncommon, even in western countries with a birth rate of a second child as high as 80% [5]. Most of the research on family transitions focused on the firstborn’s behaviour problems use different samples [5]. For example, ref. [6] used a sample from middle class families in the USA, while [7,8,9] used samples from Chinese mothers. However, second-time mothers, although possessing some experience in pregnancy, do not have an easier experience than first-time mothers [10]. Ref. [10] compared first-time mothers’ and second-time mothers’ parenting stress, marital quality, and perceptions of marital roles. He found that the transition to having a second child is similar to having the first child in terms of increased stress and declines in marital quality. However, the stress sources are different. Ref. [11] investigated the factors predicting two types of parents’ wellbeing: individual wellbeing and parenting-related wellbeing. They found that non-first-time parents were not better off than first-time parents regarding wellbeing and marital relationship quality. Ref. [12] investigated mothers’ perceptions of the transition to second-time motherhood and showed that mothers approached the birth of a second child with apprehension. However, to the best of our knowledge, little research has been performed on second-time mothers’ prenatal depression, particularly with respect to the association with firstborn children’s behaviour problems. 

The research on maternal prenatal depression is well established. A number of risk factors have been identified, including maternal anxiety, alcohol use, smoking, childhood abuse, quality of parenting during childhood, life stress, prior depression, lack of social support, social conflict, domestic violence, unintended pregnancy, relationship factors, public insurance, socio-demographic and economic factors, and so on [3,13]. However, the extant research on maternal prenatal depression did not distinguish first-time and second-time mothers. Refs. [14,15,16] have shown that multiparous women are at increased risks of prenatal depression. However, the contributing factors were not well understood. This could be due to the fact that parenting stress for second-time mothers are higher than one-child mothers [17] because of the existence of the firstborns. Therefore, the prenatal depression of second-time mothers requires further investigation. 

The association between maternal depression and adverse child outcomes is well studied in the literature [18]. Research showed that behaviour problems and developmental difficulties are more likely to occur in children with depressed mothers [6,18,19,20]. In particular, ref. [20] studied maternal and paternal trajectories of depressive symptoms and whether they can predict family risks and children’s behaviour problems after the birth of a sibling using a sample from a middle-income community in the U.S. They found that the maternal and paternal trajectories of depressive symptoms covary with firstborn children’s behaviour problems and parenting daily hassles. However, their classification of the trajectories was based on both mother’s and father’s depressive symptoms, and their focus was on the growth rate of the trajectories rather than the association between these variables. The child effect of maternal depression (i.e., how does children affect mothers’ depression) was less investigated. Both Family Systems Theory [21] and Ecological Systems Theory [22] show that the psychological and functional developments of individuals interact with their contexts, meaning that the individuals both impact and are impacted by the contexts. In our context, firstborns’ adaptation to siblinghood impacts mothers’ mental health and vice versa. The Transactional Process Model [23,24] also assumes a mutual influence between an organism and its environment. The authors of [25,26] studied the transactional relationship between child behaviour problems and maternal depressive symptoms and showed that both maternal effects and child effects were found in their samples. That is, children’s behaviour problems do affect maternal depression. However, the above research did not study the second-time mothers’ prenatal depression. Although second-time mothers are more experienced with physical changes and the pregnancy process [10], they simultaneously need to look after the firstborn child and deal with the firstborn child’s adjustment and adaptation to the transition [5,27]. Recently, ref. [28] showed that firstborn’s external behaviour problems can increase depressive symptoms. Therefore, firstborn children may be a major source of mothers’ depression during the pregnancy of a second child. 

Second-time mothers’ prenatal depression may also be impacted by daily hassles parenting, which is referred to as “minor stressors associated with parenting” [29]. It is extensively studied that parenting daily hassles affect parent’s wellbeing [29,30,31]. Refs. [29,30] reported a positive association between parenting hassles and psychological distress and wellbeing. Ref. [32] found that parenting hassles have impacts on parenting stress. For second-time mothers, looking after the first child is a very important task in their daily life, and the hassles could be stressful due to their weaker physical conditions, which may result in prenatal depression. In turn, parenting daily hassles may be impacted by the first-child’s behavioural problem [33] because behaviour problems require more attentions and care from parents [30]. Ref. [34] found that behaviour problems contribute to more daily hassles, but daily hassles did not contribute to more behaviour problems in a sample from Muslim Arab Americans. Therefore, we can derive that parenting daily hassles may play a mediation role between firstborn children’s behaviour problems and second-time mothers’ prenatal depression.

This study aims to investigate the association between firstborn children’s behaviour problems and second-time mothers’ prenatal depression in families transitioning to two children. The main questions we aim to answer are as follows: first, do firstborn children behaviour problems have any association with second-time mothers’ prenatal depression? Second, are parenting daily hassles mediating the relationship between firstborn children’s behaviour problems and second-time mothers’ prenatal depression? Based on the previous literature review, we propose the following hypotheses.

**Hypothesis** **1.** *Firstborn’s behaviour problems have positive association with second-time mother’s prenatal depression.*

**Hypothesis** **2.** *Parenting daily hassle mediates the association between firstborn’s behaviour problems and second-time mother’s prenatal depression.*

## 2. Materials and Methods

### 2.1. Participants

Participants need to meet the following criteria: (1) the firstborn child is between 2 and 5 years old; (2) no physical health problems for both the firstborn child and mother; and (3) the mother is in the third trimester of pregnancy with respect to the second child. Participants numbering 105 were recruited and asked to fill an online questionnaire with the support of research assistants, and all of the questionnaires were valid. It should be noted that we did not exclude women with previous psychiatric comorbidities from this sample, which could be a limitation of this research. The samples are from 20 provinces in China with 37.14% from Jilin Province and 1% to 9% from other provinces. Of the 105 participants, the firstborn’s average age is 48 months (M = 48.34; SD = 12.34), and second-time mother’s age ranges between 24 and 41 with an average of 33 (M = 32.83; SD = 3.75). All participants are living with their husbands and their first child with average marriage time periods being 8 years (M = 7.97; SD = 3.04). Table 1 lists the demographic variables. From Table 1, we can observe that more than 80% of mothers received college education and about half of the families have annual incomes between 100k and 500k RMB and about 42% of the families had lower than 100k RMB, which means that our sample represents a well-educated lower-income and middle-income population in China.

According to the National Bureau of Statistics of China (Households’ Income and Consumption Expenditure in 2020 (stats.gov.cn)), China’s per capita disposable income was 32,189 RMB in 2020, which means that the average household income is about 100k RMB considering that the average family size is about 3 persons. Since our samples include 43% families with income lower than 100k and half with income between 100k and 500k, it is fair to say that the sample represents a lower and middle-income population.

### 2.2. Procedures

Participants were recruited by word-of-mouth messages on Chinese social media application WeChat. First, a WeChat official account was created for the project and recruitment advertisement was posed. Second, 400 undergraduate students in the first author’s institution were briefed with recruitment requirements and asked to help disseminate information in their networks and recommend potential qualified second-time mothers. For each successful recommendation, a small red packet of RMB 30 Yuan was given. Instruction about the research was provided at the beginning of the questionnaire detailing the purpose of this research, which is to study the relationship between firstborn’s problem behaviour and second-time mother’s mental health. In addition, in the introduction section of the questionnaire, the participants were informed about the anonymous and voluntary nature of the participation, and the mother’s consent regarding the use of data in this research was obtained before answering the questions. Participants numbering 105 were recruited. The participants were selected purely based on the criteria mentioned above. Ethical approval was obtained from the first author’s research review board. Twelve trained research assistants were asked to assist the participants in filling the questionnaire online. Participants received RMB 50 Yuan for their time.

### 2.3. Measures

Most of the measures have been used in the China context [35]; thus, the Chinese version of the questionnaires were used if there was one. For those without readily available Chinese versions (Parenting Daily Hassles Scale), the researchers translated the questionnaires, which were back-translated by a professional translator to ensure the equivalence of the two versions [36].

#### 2.3.1. Children Behaviour Problems

The Child Behaviour Checklist for Ages 11/2-5 (CBCL/11/2-5) [37] is one of the most used measures for child psychology for evaluating behavioural and emotional problems in children with ages between 11/2 and 5 years. It has been translated into Chinese with tested applicability in mainland China [38].

Mothers rated 99 items about their children’s behaviour problems in the past two months with 3-point Likert scales from 0 = not true to 2 = very true. The total score was used to evaluate the overall behaviour problems. Cronbach’s α was 0.968 in this sample.

#### 2.3.2. Mother’s Prenatal Depression

The Beck Depression Inventory (BDI) [39] was used to evaluate second-time mothers’ depression. The Chinese version was reported with high internal reliability and concurrent validity in mainland China [35]. BDI is a 21-item scale and has been used in many studies including prenatal maternal depression [6]. Each item was graded from 0 to 3. In this study, we summed the item scores to create a composite score for mothers. Cronbach’s α was 0.901 in our sample.

#### 2.3.3. Parenting Daily Hassles

The Parenting Daily Hassles (PDH) Scale [31] was used to measure mothers’ perceptions about parenting daily hassles on their firstborn children. This paper uses the original version of the scale comprising 20 items that describe discrete events involving challenging child behaviour and various parenting tasks. In this study, item 7 (“Sibling arguments or fights require a ‘referee’”) and item 18 (“Difficulties in leaving kids for a night out or at school or day care”) are not applicable in China. Thus, they were removed from our questionnaire. We also removed another 3 items due to the unclear meaning. They are “2. Being nagged, whined at, complained to,” “4. The kids won’t listen and do what they are asked without being nagged,” and “20. Having to run extra errands to meet the kids’ needs.” Each item is rated for the frequency of occurrence (a 4-point scale from “rarely” to “constantly”) and the intensity at which the mothers perceive the event as a hassle (a 5-point scale from “1 = no hassle” to “5 = big hassle”). Due to the fact that the two scales are highly correlated (*r* = 0.78) [31], only the intensity score was used in this study with a sum of 15 item scores. Similarly, the daily hassle intensity was also used by [9,20] to measure parenting hassles. Cronbach’s α was 0.917 in our sample.

## 3. Results

### 3.1. Data Screening

Prior to the main analysis, the data should be screened so that the data satisfy the conditions of statistical tools. As mentioned before, all the questionnaires are valid without missing data or outliers due to the method of data collection. The most important issue for the following multivariate analysis is to check the normality of the variables. In order to perform this, we calculate skewness and kurtosis for firstborn’s behaviour problem (1.662, 4.513), second-time mother’s prenatal depression (1.240, 1.377), and parenting daily hassles (−0.029, −0.906). They are all between −2 and 2. We also conducted the Kolmogorov–Smirnov (K-S) and the Shapiro–Wilk normality tests for the three variables. Prenatal depression and behaviour problems were statistically significant (*p* < 0.001) under both the K-S and Shapiro–Wilk normality tests. Although the K-S test was not significant for parenting daily hassles (*p* = 0.191), the Shapiro–Wilk test was significant (*p* < 0.05). Since the Shapiro–Wilk test can provide better power than the K-S test [40], we can say that the three variables follow normal distribution, and we can proceed with statistical analysis on the data.

### 3.2. Descriptive Analysis

**Figure 1:**
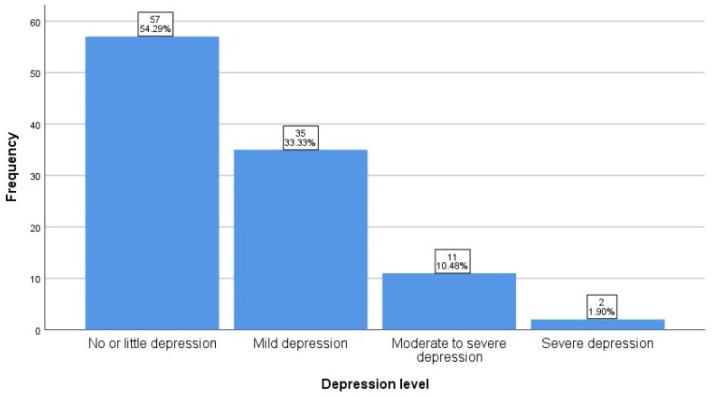
Frequency of second-time mother’s depression.

We first analyzed second-time mother’s prenatal depression. By using the suggested score ranges for mild depression (10–19), moderate to severe depression (20–30), and severe depression (31 or higher) by [41], the second-time mother’s depression levels are depicted in Figure 1. From Figure 1, we can see that about 12.4% second-time mother has moderate or severe depression, which is higher than the study in [20]. If we include the mothers with mild depression, about half (46.7%) of the second-time mothers have some degree of depressive symptoms.

We then conducted a correlation analysis among variables and descriptive statistics, which are presented in Table 2. From Table 2, we can observe that second-time mother’s depression is positively correlated with firstborn’s behaviour problems and parenting daily hassle but negatively correlated with her age. Parenting daily hassle is positively correlated with the firstborn’s behaviour problems but without significant correlation with mother’s age.

In addition, a series of ANOVA tests showed that there were no significant main effects with respect to the firstborn’s gender (*p* = 0.398), income level (*p* = 0.285), and mother’s education level (*p* = 0.159) on second-time mother’s prenatal depression.

### 3.3. Main Effects

We first tested the main effect of firstborn’s behaviour problem on second-time mother’s prenatal depression. Due to the fact that only the mother’s age was significant in the correlation analysis, we placed it in the model as a control variable. Table 3 presents the result of the regression analysis. The main effect of firstborn’s behavioural problem was significant, which indicated that the firstborn’s behavioural problem was positively associated with second-time mother’s prenatal depression (β = 0.22, *p* < 0.05).

Therefore, Hypothesis 1 “Firstborn’s behaviour problems have positive association with second-time mother’s prenatal depression” is supported.

### 3.4. Mediation Effect

**Figure 2:**
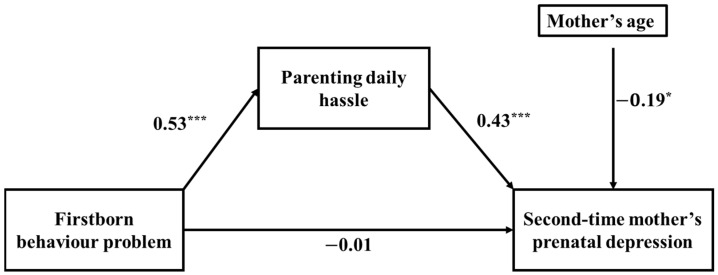
Standardized regression coefficients for the relationship between firstborn’s behaviour problems and second-time mother’s prenatal depression mediated by parenting daily hassle. * *p* < 0.05, *** *p* < 0.001.

In order to test Hypotheses 2, the standard procedure for simple mediation analysis [42] was followed by using Model 4 in the PROCESS macro for SPSS. The 95% confidence intervals (CI) for the effects were calculated by using a bootstrapping method with 5000 samples. The significance of an effect was decided by checking if the CIs contain zero or not [42]. The variable of mother’s age was entered in the final model as a control variable. The model’s output is depicted in Figure 2, which shows that the relationship between firstborn’s behaviour problems and second-time mother’s prenatal depression is mediated by parenting daily hassle.

However, the coefficient between firstborn’s behaviour problems and second-time mother’s prenatal depression was not statistically significant, meaning that the effect of firstborn’s behaviour problem was fully mediated by parenting daily hassle. The standardized regression coefficient between firstborn’s behaviour problems and parenting daily hassles was statistically significant, as was the standardized regression coefficient between parenting daily hassles and second-time mother’s prenatal depression. The standardized indirect effect on this model was (0.53) × (0.43) = 0.23. We used a bootstrapping procedure to test the significance of this indirect effect with 5000 bootstrapped samples and computed the 95% CI by determining the indirect effects at the 2.5th and 97.5th percentiles, as illustrated in Table 4. The bootstrapped unstandardized indirect effect was 0.11, and the 95% CIs ranged from 0.06 and 0.17. Therefore, we can say that the indirect effect is statistically significant.

Therefore, Hypothesis 2, “Parenting daily hassle mediates the association between firstborn’s behaviour problems and second-time mother’s prenatal depression,” was supported.

## 4. Discussion

### 4.1. Second-Time Mother’s Prenatal Depression

In this study, we found that about half of the second-time mothers have some degree of depressive symptoms, which is consistent with the results of a recent study by [1] using Chinese samples. However, we cannot say that about half of second-time mothers are depressed because the cutoff scores are not for clinical purposes [4]. If we examine the number of samples with moderate or above depressive symptoms, the proportion is 12.4%, which is in line with the literature showing the rate of prenatal depression ranging from 1.7 to 20.8 depending on measures and criteria [43]. Although most studies in the literature did not distinguish if the participants were experiencing first or second pregnancies, research shows that second-time mothers are not easier than first-time mothers [10] due to different stress sources. For second-time mothers, increased stress could result from the firstborn child. The authors of [20] reported less than 6.8% (less than 12.4% in our sample) of mothers with moderate or higher depression symptoms before the birth of second child, which is partly because their samples are community-based with middle or high income. Although our research is not for clinical purpose, the 12.4% of mothers with moderate or above depressive symptoms calls for attentions on second-time mothers’ mental health, which could be an emerging social issue after the relaxation of the birth control policy in China. Further investigations will be needed on second-time mothers’ prenatal depression.

### 4.2. The Association between Firstborn’s Behaviour Problem and Second-Time Mother’s Prenatal Depression

Our results show that firstborn’s behaviour problem contributes to second-time mother’s prenatal depression. This is new in maternal prenatal depression literature, which is mainly focused on mother’s personal characteristics such as anxiety, smoking, social support, and so on [3,13]. The reason is straightforward: Most of the existing research on maternal prenatal depression are only focused on pregnant women without considering if they have other children. However, for families transitioning to a two-child format, the firstborn’s behavioural problem is an important part of the second-time mothers’ life stress, which is a risk factor for the mother’s mental health [32]. This factor does not exist for first-time mothers. This finding is particularly useful in China’s context since the long-time “one-child policy” makes people ignore the firstborn’s impact on second-time mothers. This finding provides empirical evidence for the association between firstborn’s behaviour problem and second-time mother’s prenatal depressive symptom. One thing that should be noted is that the average age of the firstborns (4 years in this paper) is different from studies in the U.S. and Europe, with average age of 2.5 years [20]. This is due to lifting “one-child policy” in China. The older firstborns and older mothers mean more challenges for second-time mothers.

### 4.3. The Mediation Role of Parenting Daily Hassles between the Association of Firstborn Behaviour Problems and Mother’s Prenatal Depression

Our study found that children’s behaviour problems can increase parenting daily hassles, which is consistent with the literature [30]. During family transition, increased behaviour problems in the firstborn will increase parents’ parenting daily hassles because more time and effort need to be spent on the firstborn. Unlike [30], our samples were second-time mothers, which complement the existing results. We also found that parenting daily hassle was positively associated with second-time mother’s prenatal depression. Increased parenting daily hassles result in higher prenatal depressive symptoms. This is different from the results from the literature review by [13] where the reviewed five research studies did not find significant associations between daily hassles and antenatal depression. However, the daily hassles in the review include not only parenting daily hassle but also other hassles such as work hassle, time pressure, etc. [13]. Our results are in line with the literature that parenting daily hassle is a significant factor for mother’s wellbeing [29,30]. For second-time mothers, they are already very stressful due to the pregnancy [1], and incrased parenting hassles from the first child will induce more struggles, which could worsen their mental health.

Unlike most of the literature, by studying the impact of maternal depression on children behaviour problems [20,44], our study showed that firstborn’s behaviour problems contribute to mother’s prenatal depression. Different from [28], our results showed that firstborn’s behaviour problems do not directly associate with mother’s prenatal depression but via parenting daily hassles. This differs from the literature with respect to the reciprocal association between children’s behaviour problems and mothers’ depressive symptoms [25,26]. This is because our study was investigating the relationship between firstborn’s behaviour problems and second-time mother’s prenatal depression, which is different from the literature.

The findings should be used cautiously due to the limitations of the study. First, the data used in this research were all reported by mothers. The shared method variance and single-reporter bias may impact the results of this research. There is evidence that different informants report differently on children’s behaviour problems [45]. However, this limitation could be avoided in future research by using both mothers’ and fathers’ reports on children behaviour problems and averaging the two scores to form a composite score [6]. Second, as the transactional process model stated, the association between children behaviour problems and maternal depression is bidirectional and the influence is reciprocal [20,25]. This research only focused on the single direction from firstborn’s behaviour problems to mothers’ prenatal depression during transitioning to siblinghood. Investigating the reciprocal relationship between firstborn’s behaviour problems and mothers’ depression, using longitudinal data will be our next step. Third, our samples are well educated and have decent income in the China context. The findings in this paper may not be generalized to other contexts. Some research shows that education level negatively associates with the willingness to have a second child [46]. Therefore, studying the less educated population would be a future research direction. Moreover, we did not distinguish whether the second-time mother had previous psychiatric comorbidities, which may be a bias for our results. Additional research could be performed to address this limitation. Another possible research direction could be exploring the impact of COVID-19 pandemic on the second-time mothers’ mental health since research has shown that the pandemic has significant impacts on the wellbeing of pregnant women [47].

## 5. Conclusions

This study contributes to our understanding of the relationship between firstborns’ behavioral problems and second-time mothers’ prenatal depression. The results emphasize the need to investigate second-time mothers’ prenatal depression, which has great implications in China because a surge of second-time pregnancies was observed after the start of “two-child policy.” In particular, firstborn behavioral problems play an important role on second-time mother’s prenatal depression indirectly via parenting daily hassles. This implies that interventions could be put in place to reduce parenting daily hassles. One possible method is to obtain more child care support for the firstborns through either family members or integrated early childhood services.

## Figures and Tables

**Table 1:** Demographic variables (**N** = 105).

Variable	Value	Number	Percentage
Firstborn gender	Boy	45	42.86%
	Girl	60	57.14%
Household income (RMB)	Below 100k	44	41.9%
	100k–200k	41	39.05%
	200k–500k	14	13.33%
	Above 500k	6	5.71%
Mother’s Education	Primary school and below	0	0%
	Secondary school	12	11.43%
	High school	17	16.19%
	College	20	19.05%
	Undergraduate	35	33.33%
	Postgraduate (Master’s and PhD)	21	20%
Firstborn’s gender	Boy	45	42.9%
	Girl	60	57.8%
Working status	Full-time work	64	58.72%
	Part-time work	12	11.01%
	No work	33	30.28%
Firstborn’s main carer	Myself	56	51.38%
	My husband	2	1.83%
	My husband’ parent/s	28	25.69%
	My parent/s	19	17.43%
	Baby sitter	4	3.67%
	Other	0	0%

**Table 2:** Correlation coefficients and descriptive statistics of variables (**N** = 105).

Variables	1	2	3	4
1. Prenatal depression	1			
2. Behaviour problems	0.27 **	1		
3. Daily hassle	0.46 **	0.54 **	1	
4. Mother age	−0.26 **	−0.23 *	−0.17	1
Mean	10.50	20.07	35.39	32.72
Std	8.42	17.38	11.28	3.82

* *p* < 0.05, ** *p* < 0.01.

**Table 3:** Regression of second-time mother’s prenatal depression (**N** = 105).

Variables	*R* ^2^	*B* (*SE*)	*β*
Constant	0.11	23.33 (7.22)	
Mother’s age		−0.46 (0.21)	−0.21 *
Firstborn’s behaviour problem		0.11 (0.05)	0.22 *

* *p* < 0.05.

**Table 4:** Effects in the mediation model.

	Effect	SE	*p*	95% CIs	
				Lower CI	Upper CI
Total effect	0.11	0.05	0.02	0.015	0.20
Direct effect	−0.00	0.051	0.93	−0.10	0.10
Indirect effect	0.11	0.03	n.a.	0.06	0.17
Standardized indirect effect	0.23	0.06	n.a.	0.12	0.35

Note: Bootstrap sample size: 5000.

## Data Availability

The dataset in this research can be obtained upon request.

## References

[1] Hu Y., Wang Y., Wen S., Guo X., Xu L., Chen B., Chen P., Xu X., Wang Y. (2019). Association between social and family support and antenatal depression: A hospital-based study in Chengdu, China. BMC Pregnancy Childbirth.

[2] Woody C.A., Ferrari A.J., Siskind D.J., Whiteford H.A., Harris M.G. (2017). A systematic review and meta-regression of the prevalence and incidence of perinatal depression. J. Affect. Disord..

[3] Biaggi A., Conroy S., Pawlby S., Pariante C.M. (2016). Identifying the women at risk of antenatal anxiety and depression: A systematic review. J. Affect. Disord..

[4] Pearson R.M., Carnegie R.E., Cree C., Rollings C., Rena-Jones L., Evans J., Stein A., Tilling K., Lewcock M., Lawlor D.A. (2018). Prevalence of prenatal depression symptoms among 2 generations of pregnant mothers: The Avon Longitudinal Study of Parents and Children. JAMA Netw. Open.

[5] Volling B.L. (2012). Family transitions following the birth of a sibling: An empirical review of changes in the firstborn’s adjustment. Psychol. Bull..

[6] Volling B.L., Gonzalez R., Oh W., Song J.-H., Yu T., Rosenberg L., Kuo P.X., Thomasin E., Beyers-Carlson E., Safyer P. (2017). Developmental trajectories of children’s adjustment across the ransition to siblinghood: Pre-birth predictors and sibling outcomes at one year. Monogr. Soc. Res. Child. Dev..

[7] Chen B.-B., Wang Y., Liang J., Lian T. (2016). And baby makes four: Biological and psychological changes and influenctial factors of firstborn’s adjustment to transition to siblinghood. Adv. Psychol. Sci..

[8] Chen B.-B., Han W., Wang Y., Sui Y., Chen Z., Wan L. (2018). The reaction of firstborn children to a sibling before the birth: The role of the time at which they are told about the mother’s pregnancy and their effortful control. J. Reprod. Infant Psychol..

[9] Chen B.-B. (2018). The relationship between Chinese mothers’ parenting stress and sibling relationships: A moderated mediation model of maternal warmth and co-parenting. Early Child. Dev. Care.

[10] Krieg D. (2007). Does motherhood get easier the second-time around? Examining parenting stress and marital quality among mothers having their first or second child. Parenting.

[11] Ketner S.L., Gravesteijn C., Verschuur M.J. (2019). Transition to parenthood: It does not get easier the next time. Exploring ways to support well-being among parents with newborns. J. Fam. Soc. Work.

[12] Chapman J.K., Hart S.L. (2017). The transition from mother-of-one to mother-of-two: Mothers’ perceptions of themselves and their relationships with their firstborn children. Infant Ment. Health J..

[13] Lancaster C.A., Gold K.J., Flynn H.A., Yoo H., Marcus S.M., Davis M.M. (2010). Risk factors for depressive symptoms during pregnancy: A systematic review. Am. J. Obstet. Gynecol..

[14] Abuidhail J., Abujilban S. (2013). Characteristics of Jordanian depressed pregnant women: A comparison study. J. Psychiatr. Ment. Health Nurs..

[15] Golbasi Z., Kelleci M., Kisacik G., Cetin A. (2010). Prevalence and correlates of depression in pregnancy among Turkish women. Matern. Child. Health J..

[16] Redshaw M., Henderson J. (2013). From antenatal to postnatal depression: Associated factors and mitigating influences. J. Women Health.

[17] Hong X., Liu Q. (2019). Parenting stress, social support and parenting self-efficacy in Chinese families: Does the number of children matter?. Early Child. Dev. Care.

[18] Goodman S.H., Rouse M.H., Connell A.M., Broth M.R., Hall C.M., Heyward D. (2011). Maternal depression and child psychopathology: A meta-analytic review. Clin. Child. Fam. Psychol. Rev..

[19] Goodman S.H. (2007). Depression in mothers. Annu. Rev. Clin. Psychol..

[20] Volling B.L., Yu T., Gonzalez R., Tengelitsch E., Stevenson M.M. (2018). Maternal and paternal trajectories of depressive symptoms predict family risk and children’s emotional and behavioral problems after the birth of a sibling. Dev. Psychopathol..

[21] Minuchin P. (1985). Families and individual development: Provocations from the field of family therapy. Child. Dev..

[22] Bronfenbrenner U. (1979). Contexts of child rearing: Problems and prospects. Am. Psychol..

[23] Sameroff A. (1975). Transactional Models in Early Social Relations. Hum. Dev..

[24] Sameroff A. (2009). The Transactional Model of Development: How Children and Contexts Shape Each Other.

[25] Heinz A.L. (2016). Kids Can Screw Up Their Parents, too: An Analysis of the Reciprocal Influences between Maternal Depressive Symptoms and Child Problem Behaviors from Child Age 2 to 15. Ph.D. Dissertation.

[26] Gross H.E., Shaw D.S., Moilanen K. (2008). Reciprocal associations between boys’ externalizing problems and mothers’ depressive symptoms. J. Abnorm. Child Psychol..

[27] Volling B.L. (2005). The transition to siblinghood: A developmental ecological systems perspective and directions for future research. J. Fam. Psychol..

[28] Volling B.L., Tan L., Gonzalez R., Bader L.R., Kuersten-Hogan R., McHale J. (2021). Coming together or falling apart: Coparenting the first child while expecting the second. Prenatal Family Dynamics: Couple and Coparenting Relationships during and Postpregnancy.

[29] Crnic K.A., Booth C.L. (1991). Mothers’ and fathers’ perceptions of daily hassles of parenting across early childhood. J. Marriage Fam..

[30] Creasey G., Reese M. (1996). Mothers’ and fathers’ perceptions of parenting hassles: Associations with psychological symptoms, nonparenting hassles, and child behavior problems. J. Appl. Dev. Psychol..

[31] Crnic K.A., Greenberg M.T. (1990). Minor parenting stresses with young children. Child. Dev..

[32] BeLue R., Halgunseth L.C., Abiero B., Bediako P. (2015). Maternal health status and parenting stress in low-income, ethnic-minority mothers of children with conduct disorder problems: The role of daily parenting hassles. J. Racial Ethn. Health Disparities.

[33] Serbin L.A., Kingdon D., Ruttle P.L., Stack D.M. (2015). The impact of children’s internalizing and externalizing problems on parenting: Transactional processes and reciprocal change over time. Dev. Psychopathol..

[34] Aroian K.J., Templin T.N., Hough E.S. (2016). Daily hassles, mother–child relationship, and behavior problems in Muslim Arab American adolescents in immigrant families. Cult. Divers. Ethn. Minor. Psychol..

[35] Tang C.S.K., Wilson J.P. (2007). Assessment of PTSD and psychiatric comorbidity in contemporary Chinese societies. Cross-Cultural Assessment of Psychological Trauma and PTSD.

[36] Pena E. (2007). Lost in translation: Methodological considerations in cross-cultural research. Child. Dev..

[37] Achenbach T.M., Rescorla L.A. (2000). Manual for the ASEBA Preschool Forms & Profiles.

[38] Liu J., Cheng H., Leung P.W.L. (2010). The Application of the preschool child behavior checklist and the caregiver–teacher report form to Mainland Chinese Children: Syndrome structure, gender differences, country effects, and inter-informant agreement. J. Abnorm. Child. Psychol..

[39] Beck A.T., Ward C.H., Mendelson M., Mock J., Erbaugh J. (1961). An inventory for measuring depression. Arch. Gen. Psychiatry.

[40] Ghasemi A., Zahediasl S. (2012). Normality tests for statistical analysis: A guide for non-statisticians. Int. J. Endocrinol. Metab..

[41] Kendall P.C., Hollon S.D., Beck A.T., Hammen C.L., Ingram R.E. (1987). Issues and recommendations regarding use of the beck depression inventory. Cogn. Ther. Res..

[42] Hayes A.F. (2013). Introduction to Mediation, Moderation, and Conditional Process. Analysis: A Regression-Based Approach.

[43] Guyon-Harris K., Huth-Bocks A., Lauterbach D., Janisse H. (2015). Trajectories of maternal depressive symptoms across the birth of a child: Associations with toddler emotional development. Arch. Women Ment. Health.

[44] Vänskä M., Punamäki R.-L., Tolvanen A., Lindblom J., Flykt M., Unkila-Kallio L., Tiitinen A., Repokari L., Sinkkonen J., Tulppala M. (2011). Maternal pre- and postnatal mental health trajectories and child mental health and development: Prospective study in a normative and formerly infertile sample. Int. J. Behav. Dev..

[45] Grietens H., Onghena P., Prinzie P., Gadeyne E., Van Assche V., Ghesquiere P., Hellinckx W. (2004). Comparison of mothers’, fathers’, and teachers’ reports on problem behavior in 5- to 6-year-old children. J. Psychopathol. Behav. Assess..

[46] Tian L.F., Rong T.H., Zhang X.Y., Sun Q., Zhang J.C., Zhang H.D., Gao J.W. (2017). Impact factors on willingness to have a second child for rural residence under the “universal two-child” policy. Popul. Dev..

[47] Biviá-Roig G., La Rosa V.L., Gómez-Tébar M., Serrano-Raya L., Amer-Cuenca J.J., Caruso S., Commodari E., Barrasa-Shaw A., Lisón J.F. (2020). Analysis of the impact of the confinement resulting from COVID-19 on the lifestyle and psychological wellbeing of Spanish pregnant women: An internet-based cross-sectional survey. Int. J. Environ. Res. Public Health.

